# Analysis of the genetic variance of fibre diameter measured along the wool staple for use as a potential indicator of resilience in sheep

**DOI:** 10.1186/s12711-024-00924-4

**Published:** 2024-08-06

**Authors:** Erin G. Smith, Dominic L. Waters, Samuel F. Walkom, Sam A. Clark

**Affiliations:** 1 School of Environmental and Rural Science, University of New England, Armidale, NSW 2351 Australia; 2 Animal Genetics and Breeding Unit, University of New England, Armidale, NSW 2351 Australia

## Abstract

**Background:**

The effects of environmental disturbances on livestock are often observed indirectly through the variability patterns of repeated performance records over time. Sheep are frequently exposed to diverse extensive environments but currently lack appropriate measures of resilience (or sensitivity) towards environmental disturbance. In this study, random regression models were used to analyse repeated records of the fibre diameter of wool taken along the wool staple (bundle of wool fibres) to investigate how the genetic and environmental variance of fibre diameter changes with different growing environments.

**Results:**

A model containing a fifth, fourth and second-order Legendre polynomial applied to the fixed, additive and permanent environmental effects, respectively, was optimal for modelling fibre diameter along the wool staple. The additive genetic and permanent environmental variance both showed variability across the staple length trajectory. The ranking of sire estimated breeding values (EBV) for fibre diameter was shown to change along the staple and the genetic correlations decreased as the distance between measurements along the staple increased. This result suggests that some genotypes were potentially more resilient towards the changes in the growing environment compared to others. In addition, the eigenfunctions of the random regression model implied the ability to change the fibre diameter trajectory to reduce its variability along the wool staple.

**Conclusions:**

These results show that genetic variation in fibre diameter measured along the wool staple exists and this could be used to provide greater insight into the ability to select for resilience in extensively raised sheep populations.

**Supplementary Information:**

The online version contains supplementary material available at 10.1186/s12711-024-00924-4.

## Background

Animals experience many disturbances in their internal and external environment which can compromise their health, welfare and productivity. For these reasons, there is growing interest in selecting animals that have improved resilience to these disturbances. Resilience is defined as the capacity of an animal to be minimally affected by disturbance or to rapidly recover from a disturbance [1]. In intensive livestock and aquatic species, resilience has been measured using the variable rate of resource allocation into production tissues and structures such as milk yield, growth and egg production using high-frequency phenotypic measures [2]. Deviation from normal patterns of production are inferred to represent poor resilience whereby resources are diverted through other metabolic pathways such as defence and repair. Conversely, animals with greater uniformity of these productive outputs across time are suggested to have higher resilience because they are not, or are less, affected by disturbances in their internal and external environment. Having animals that are genetically well adapted to their production environment using such measures is potentially beneficial as it has been shown to reduce the likelihood of negative health and welfare outcomes, which is of growing importance to consumers. However, more work is required to quantify resilience in extensively raised livestock such as sheep, in the absence of high-frequency phenotyping methods [3]. The importance of resilience in extensively raised livestock is paramount due to their exposure to disturbances such as poor nutrition, diseases, harsh and variable climates and the provision of lower levels of management interventions.

Fibre diameter variation measured along a wool staple (bundle of fibres) has recently been proposed as a suitable data source for quantifying resilience in extensively raised sheep [4–6]. During the anagen (growth) phase of wool growth, conditions prevailing in the internal and external environments influence both the rate of fibre elongation and the diameter of the fibres produced [7, 8]. The variability in the incremental growth patterns of wool is associated with known stress events such as nutrition, diseases and climate [9–11]. The lag time between when these stresses are present and the appearance in the fibre diameter depends on the type of environmental stress -or example, acute health conditions (e.g., flystrike) can appear within several days, but gradual stresses such as nutritional changes can take around 3 weeks to appear in the fibre [12]. Previous studies have established genetic variation in the uniformity of fibre diameter over the year using simple trait definitions such as along fibre coefficient of variation and absolute change in fibre diameter [6, 13]. This suggested that it might be possible to select animals that have less variation in fibre diameter and this could be indicative of greater resilience. However, the trait definition of resilience from this data source remains difficult to establish, particularly because it involves dynamic features such as the rate of response and recovery from disturbance. A further complication is that the full response to a disturbance is typically expressed across multiple measurements in the fibre diameter profile and therefore requires a sophisticated approach to be able to properly characterise the changes in fibre diameter across the growing period (equivalent to the length of the wool staple) [6].

Fibre diameter profiles can be regarded as repeated records of fibre diameter measured across the wool staple length grown within a specified time period. Animal breeding has several methods for accounting for the genetic variation in repeated records over time, such as the repeatability model, multi-trait model and the fitting of curves to the phenotypic values across time points. The latter is possibly most appropriate for fibre diameter profiles, as the fitting of curves accounts for the changes in genetics and environmental variation over the staple length, where the staple length can be used to represent the duration of exposure to disturbances in a given environment. Random regression models are among the most common methods to facilitate the estimation of genetic parameters for traits that change over a trajectory [14, 15]. The models fit the average curve for a subpopulation (e.g., contemporary group) and animal-specific curves describe deviations from the groups’ average curves. The regression coefficients of the random regression model indicate how the additive genetic effect of an animal changes, where animals with larger changes in effect indicate an animal that is influenced by disturbances [2]. The objective of this study was to investigate the variability of fibre diameter over the length of wool staple using random regression models to potentially describe the resilience of sheep toward environmental disturbances.

## Methods

This study was separated into two parts. In the first, the fit of the random regression models based on different orders of Legendre polynomials was tested using model selection criteria and visual inspection. The second part of this study examined the parameters from the optimal model to explain how the genetic variation in fibre diameter changes along the staple length and how this can potentially be used to quantify the resilience of sheep.

### Dataset

**Fig. 1:**
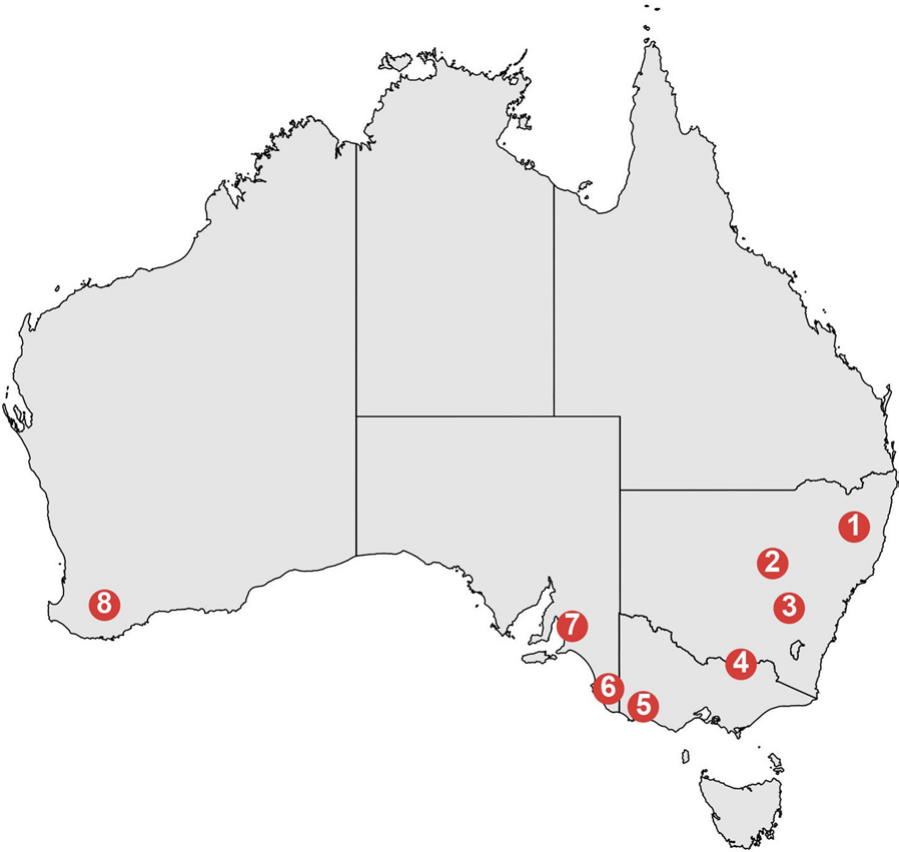
Map of the Information Nucleus Flocks located across Australia

The data used in this study originated from the Australian Sheep Cooperative Research Centre Information Nucleus Flocks [16, 17]. These flocks were situated in eight distinct environments, each representative of Australia's major wool and sheep production areas (Fig. 1). Specifically, Flock 1 was located in a summer-dominant rainfall zone, while Flocks 2 and 3 experienced even seasonal rainfall. Flocks 4 and 5 were characterised by temperate (winter) rainfall patterns, whereas Flocks 6, 7, and 8 exhibited a Mediterranean rainfall pattern. Further details regarding the specific locations of these flocks can be found in [18]. The animals used in this study were restricted to the Merino and Poll Merino animals (55% Merino and 45% Poll Merino) recorded for fibre diameter profiles at two years of age (mean 19 months, min 16, max 23). Wool characteristics measured at this age are typically well correlated to measurements taken at other life stages [19]. The data set analysed includes 4181 animals (72% female and 28% male) recorded across five years between 2009 to 2013. Each year wool samples were collected from the midside region of the animal (13th rib on the left-hand side) approximately one month before shearing, meaning the wool staples measured were approximately 11 months of wool growth. The midside location has been shown to be representative of the variation across the entire fleece (reviewed by Scobie et al. [19]). The fibre diameter of the greasy wool sample was measured at 5 mm increments along the staple at a commercial wool testing laboratory according to the standard protocol to produce the fibre diameter profile measurements. The mean fibre diameter at each location (increment) along the staple was taken as an average of the fibres measured (~ 1000 fibres). A full description of the instrumentation and methodology used to measure fibre diameter profiles are provided by [20, 21].

The fibre diameter profile records were standardised to a common length scale to accommodate differences in staple length between animals. This calculation was based on the proportion of the animals’ maximum raw staple length, where the standardised values ranged between the interval 0 and 100 [6, 11]. This standardisation assumes that the fibre growth occurs at a linear rate over the staple length and is made in the absence of other information used to determine the rate of fibre elongation (i.e., dye banding or isotope analysis). This operation was used to improve the overall graphical representation of the results presented later in the manuscript. The distribution of records along the staple length and the number of records per animal are shown in Additional file 1: Figure S1. The mean number of fibre diameter records per animal was 19.8 (min 8 records and max 27 records). Animals with fewer than 8 repeated records (equivalent to less than 40 mm of length) were removed from the analysis. The fibre diameter records were also standardised to the population mean fibre diameter to have a mean of 0 and a standard deviation of 1, where the mean was calculated from all records in the analysis. The raw mean fibre diameter of the population was 18.5 µm (min 13.6 µm, max 25.3 µm) with the mean fibre diameter at each flock shown in Additional file 2: Table S1. Once the animal's fibre diameter and staple length records were standardised, animals with standardised fibre diameter records greater than 3 SD from the mean of their respective flock-year grouping were removed from the dataset. The mean number of animals per flock-year group was 110, with 38 flock-year groups in total. A full description of the number of animals in each flock-year is provided in Additional file 2, Table S1. An example of the fibre diameter profile curves is provided in Additional file 3: Figure S2. Together a total of 80,791 fibre diameter records were included in the analysis.

All animals included in the analysis had known sire and dam. The animals were born from artificial insemination from 176 sires and the pedigree was verified from DNA parentage results. The pedigree data was traced back three generations or to an unknown parent group, with 10,181 animals present in the pedigree. Genetic groups were allocated according to the method of Swan et al. [22] and were used in the analysis to account for the varying levels of pedigree information in the base animals and the differing ewe bases within each of the Information Nucleus Flocks. On average, each sire had 24.3 progeny, ranging from a minimum of 10 to a maximum of 50. These progeny were distributed across an average of 5 flocks, with a minimum of 2 and a maximum of 8 flocks. The number of progeny per flock per sire ranged from 3 to 17, with an average of 5 sire progeny per flock, where 12 sires had progeny distributed across all 8 flocks.

### Statistical analysis

To estimate the variance and covariance components of standardised fibre diameter over the staple length, a random regression model using Legendre polynomials was used to fit the systematic fixed effects, the fixed regression of standardised fibre diameter for each flock-year, additive genetic, permanent environmental and residual effects. The use of Legendre polynomials requires that the staple length intervals be redefined on the interval of − 1 to + 1; however, the results are back-transformed to the 0–100 mm scale to enhance the interoperability of the results. The model equation was based on [14] and [15], and the general form:

$${y}_{itlj}={F}_{l}+ \sum_{m=0}^{{q}_{1}}{\varnothing }_{tm}{\beta }_{jm }+ \sum_{m=0}^{{q}_{2}}{\varnothing }_{tm}{a}_{im}+ \sum_{m=0}^{{q}_{3}}{{\varnothing }_{tm}pe}_{im}+{e}_{itlj},$$

where ${y}_{itlj}$ was the standardised fibre diameter record of animal $i$ taken at the staple length $t$ belonging to the $l$th fixed factor and the *j*th flock-year; ${F}_{l}$ represents the fixed effects that are independent of the time scale of the observations and were a concatenation of wool type (3 levels, fine, medium and strong, described by [23]), management group (66 levels) and sex (2 levels). ${\beta }_{jm}$ are the *m*th fixed regression coefficient for the mean curve of the* j*th flock year; ${a}_{im}$ and ${pe}_{im}$ are the *m*th random regression coefficient of the additive genetic and permanent environmental effects, respectively for the *i*th animal. In this model these coefficients are assumed to follow $a \sim N\left(0, {\mathbf{K}}_{\mathbf{a}}\otimes \mathbf{A}\right)$ and $pe \sim N\left(0, {\mathbf{K}}_{\mathbf{p}\mathbf{e}}\otimes \mathbf{I}\right)$, where ${\mathbf{K}}_{\mathbf{a}}$ and ${\mathbf{K}}_{\mathbf{p}\mathbf{e}}$ are the covariance matrix among the additive genetic effects and permanent environmental effects, respectively; $\mathbf{A}$ represents the additive genetic relationship matrix (which included the genetic groups); $\mathbf{I}$ represents the identity matrix and ⊗ denotes the Kronecker product. ${\varnothing }_{tm}$ is the covariate of the *m*th Legendre polynomial at staple length $t;$ ${q}_{1}$, ${q}_{2}$, and ${q}_{3}$ are the orders of fit of Legendre polynomials for the fixed, additive and permanent environmental effects, respectively. ${e}_{itlj}$ are residual effects which were assumed to be either homogenous, heterogenous for five classes of the staple length (0–19, 20–39, 40–59, 60–79, 80–100 mm) or defined by the flock-year term (38 classes). The residual effects are assumed to follow $e \sim N\left(0, \mathbf{R}\right)$, where **R** refers to a matrix of residual variance structure (homogeneous or heterogeneous). All analyses were carried out using ASReml-R [24].

### Model comparison

The random regression models were built by first establishing the order of Legendre polynomial applied to the fixed regression of fibre diameter over the staple length for each flock-year combination. The fixed regression was modelled at the flock-year level to capture the variability in fibre diameter over the staple length owing to factors associated with rainfall, pasture growth, management and shearing time. The significance of the polynomials applied to the flock-year term was tested using the partial F-test [25] and the Bayesian Information Criteria (BIC) [26]. The F-statistic was estimated by dividing the sum of the coefficients estimated by the sum of their standard errors, and then squaring the resulting term. The critical values were determined in R [27] using the numerator and denominator degrees of freedom provided by the ASReml-R output. The level of significance was set at p = 0.05 to compare sequential models in the partial F test. The BIC was calculated as; $\text{BIC}= -2\text{ logL}+\text{p log}\left(\text{N}-\text{r}\right),$ where $\text{p}$ was the number of parameters to be estimated, $\text{N}$ was the total number of observations, $\text{r}$ was the rank of the incidence matrix of fixed effects in the model and $\text{logL}$ was the logarithm of the restricted maximum likelihood function. The order of fit was further checked using visual inspection of the residual versus fitted values plots, where the residuals should be randomly scattered around the central zero line. Together the order of Legendre polynomial for the fixed regression described the ‘overall’ or ‘average’ fibre diameter curves of each flock-year. The same order applied to the fixed curve was used for different random effects models which makes the residual maximum likelihoods (REML) directly comparable. Individual animals were allowed to deviate from their flock-year curves and therefore have different coefficients of fibre diameter curves at both the genetic and the permanent environment levels using different orders of Legendre polynomial applied to the random effects.

The random effects included in the model were the additive genetic and permanent environmental effects. The fit of the additive genetic effect was tested using Legendre polynomials of orders one to four based on preliminary testing (results not shown). The permanent environmental effect which captures the environmental differences between repeat records of an individual was also modelled using first and second order Legendre polynomials.

The residual variance was modelled in three forms; homogenous (ho); heterogeneous (he) defined by five sections across the staple length (0–19, 20–39, 40–59, 60–79, 80–100 mm) which were determined by graphical examination of fibre diameter changes; and heterogeneous residual for each flock-year (fy). In the latter case, the differences in residuals may be related to the environmental conditions, sample error and measurement error variances at the flock-year level. After finalising the model fit of the fixed regression curves, this study required the analysis of 24 random regression models.

The preferred order of Legendre polynomial fitted to the additive and permanent environmental effects was identified based on two criteria; BIC [26] and the Penalising Adaptive Likelihood values (PAL) [28]. These criteria were used because they each apply stringent penalties to models of higher complexity. The PAL values were calculated as; $\text{PAL}= -2{\text{logL}}_{\text{n}}+\text{nlog}\left(\widetilde{\text{n}}\right) \frac{\text{ln}({\text{r}}_{\text{n}}+1)}{\text{ln}({\uprho }_{\text{n}}+1)}$, where $\text{n}$ was the number of parameters in the model, $\widetilde{\text{n}}$ was the largest number of parameters for the model within the set of model being considered, ${\text{r}}_{\text{n}}=2{\text{lnL}}_{\text{n}-1}-2{\text{lnL}}_{1}$, and ${\uprho }_{\text{n}}= 2{\text{lnL}}_{\widetilde{\text{n}}}-2{\text{lnL}}_{\text{n}-1}$. Where ${\text{r}}_{\text{n}}$ and ${\uprho }_{\text{n}}$ were generalised likelihood ratios between model ${\text{M}}_{\text{n}-1}$ and the reduced model ${\text{M}}_{1}$ or the complete model ${\text{M}}_{{\tilde{n}}}$, respectively. The PAL values have been previously advocated as the best criterion for polynomial order selection in random regression models according to [29] due to its ability to assess both consistency and efficiency. The model fitted to the data with the smallest values of BIC and PAL was regarded as optimal and was used to estimate the variance components and genetic parameters.

### Variance components and genetic parameter estimation

Once the optimal model was identified, estimates of the additive genetic variance for standardised fibre diameter at different staple lengths were obtained using; ${\widehat{\upsigma }}_{\text{a}t }^{2}={\mathbf{t}}_{{\varvec{t}}} {\widehat{\mathbf{K}}}_{\mathbf{a}} {\mathbf{t}}_{{\varvec{t}}}^{\prime}$, where ${\widehat{\mathbf{K}}}_{\mathbf{a}}$ was the estimated covariance matrix of random regression coefficients for the additive genetic effects and ${\mathbf{t}}_{t}$ are the row vectors of Legendre covariates evaluated at staple length $t$. The estimates of permanent environmental variances (${\widehat{\upsigma }}_{\text{pe}}^{2}$) was calculated using the same approach replacing ${\widehat{\mathbf{K}}}_{\mathbf{a}}$ with ${\widehat{\mathbf{K}}}_{\mathbf{p}\mathbf{e}}$, where ${\widehat{\mathbf{K}}}_{\mathbf{p}\mathbf{e}}$ was the estimated covariance matrix of random regression coefficients for the permanent environmental effects. The estimates of residual variances (${\widehat{\upsigma }}_{\text{e}j}^{2}$) were considered to change according to the flock-year, which was shown to be the preferred residual variance structure in the optimal model. The heritability of standardised fibre diameter at staple length $t$ in the $j$th flock-year was then estimated as; ${\widehat{\text{h}}}_{tj}^{2}= \frac{{{\widehat{\upsigma }}_{\text{a}t }^{2}}}{{{\widehat{\upsigma }}_{\text{a}t }^{2} + {\widehat{\upsigma }}_{\text{pe}t }^{2}+ {\widehat{\upsigma }}_{\text{e}j}^{2} }} .$ Standard errors for the variance components and heritabilities were estimated using a Taylor series expansion described by [30]. The genetic correlations of standardised fibre diameter between staple length increment was estimated using; ${\widehat{\text{r}}}_{\text{g}}= \frac{{\widehat{\upsigma }}_{\text{a}({t},{t}x)}}{\sqrt{ {{\widehat{\upsigma }}_{\text{a}({t}) }^{2}} {{\widehat{\upsigma }}_{\text{a}({t}x) }^{2}}}}$, where ${\widehat{\upsigma }}_{\text{a}\left(t,tx\right)}$ was the estimate of the covariance of additive genetic effects between pairs of staple length $t$ and $tx$; ${\widehat{\upsigma }}_{\text{a}({t})}^{2}$ and ${\widehat{\upsigma }}_{\text{a}({t}x)}^{2}$ are the additive genetic variances at staple lengths $t$ and $tx$, respectively.

The Estimated Breeding Values (EBV) for animal $i$ were estimated at the staple length $t$ from the regression coefficients using; ${\text{EBV}}_{it }= {{\widehat{\mathbf{u}}}_{{\varvec{i}}}\mathbf{t}}_{{\varvec{t}}}^{\prime},$ where ${\widehat{\mathbf{u}}}_{{\varvec{i}}}$ was a vector with the estimated breeding values for the regression coefficients defining the change in fibre diameter for a given animal $i.$ Sires which showed greater change in their breeding values plotted along the staple length were hypothesised to show greater sensitivity to environmental variation and therefore may be interpreted as being less resilient. To quantify the amount of re-ranking, Spearman rank correlation between sire EBV were calculated between five lengths (0, 25, 50, 75 and 95 mm) as; $\uprho =1- \frac{6 \sum {\text{d}}_{t}^{2}}{\text{n}\left({\text{n}}^{2}-1\right)},$ where ${\text{d}}_{t}$ was the difference between sire EBV rank at staple length $t$ and ${t}_{x}$ and $\text{n}$ was the number of sires.

To determine how the pattern of the genetic variation of fibre diameter changes over the staple length $t$, the eigenfunctions of the additive genetic covariance matrix were calculated by the following expression [31]; ${\psi }_{t}= {\sum }_{d=0}^{{q}_{2}}{({c}_{{\uppsi }_{f}})}_{d}{\mathbf{t}}_{{\varvec{t}}},$ where ${q}_{2}$ was the order of Legendre polynomial applied to model the additive genetic effects, ${({c}_{{\uppsi }_{f}})}_{d}$ was the $d$th element of the $f$th eigenvector of ${\widehat{\mathbf{K}}}_{\mathbf{a}}.\boldsymbol{ }$ Each eigenfunction is associated with an eigenvalue which is proportional to the genetic variation corresponding to that eigenfunction. These functions indicate the most probable direction of selection and whether antagonism between standardised fibre diameter is present at different staple lengths.

## Results

### Model fit and comparison

A Legendre polynomial of order five was shown to be the most appropriate to model the average curves for each flock-year based on partial F test results (see Additional file 4: Table S2). The model fit was further confirmed by examining the mean of the residuals using residual versus fitted plots (see Additional file 5: Figure S3). The fitting of lower-order Legendre polynomials was shown to underfit the data as shown by the discernible pattern among the residuals.

For the random effects, the model fit improved as the order of the Legendre polynomial increased for both the additive and permanent environmental effects (Table 1). According to the selection criteria, the optimal model contained a fourth and second order Legendre polynomial for the additive genetic and permanent environmental effects, respectively, and fitted separate residual variances for each flock-year (see model LP.4.2.Rfy in Table 1). This indicated that there were significant benefits in accounting for heterogenous residual variances among flock-years. The following results are based on the preferred model LP.4.2.Rfy.

**Table 1:** Model selection criteria of random regression models with different orders of Legendre polynomials for the additive and permanent environmental effect

Model^a^	Number of parameters	Selection criteria^b^	
		BIC	PAL
LP.1.1.Rho	7	− 88,243	–
LP.1.2.Rho	10	− 99,946	− 99,787
LP.2.1.Rho	10	− 99,757	− 100,028
LP.2.2.Rho	13	− 99,944	− 100,066
LP.3.1Rho	14	− 106,498	− 106,614
LP.3.2Rho	17	− 107,378	− 107,530
LP.4.1.Rho	19	− 113,742	− 113,624
LP.4.2.Rho	22	− 112,208	− 113,896
LP.1.1Rhe	11	− 88,705	–
LP.1.2Rhe	14	− 100,655	− 100,502
LP.2.1.Rhe	14	− 100,464	− 100,767
LP.2.2.Rhe	17	− 100,655	− 100,834
LP.3.1.Rhe	18	− 108,917	− 109,063
LP.3.2.Rhe	21	− 110,034	− 110,661
LP.4.1.Rhe	23	− 113,972	− 114,038
LP.4.2.Rhe	26	− 114,214	− 114,107
LP.1.1.Rfy	44	− 92,319	–
LP.1.2.Rfy	47	− 103,252	− 103,688
LP.2.1.Rfy	47	− 103,452	− 103,791
LP.2.2.Rfy	50	− 103,834	− 103,891
LP.3.1.Rfy	51	− 110,456	− 110,828
LP.3.2.Rfy	54	− 111,518	− 111,949
LP.4.1.Rfy	56	− 115,817	− 116,223
LP.4.2.Rfy	59	*− 116,015*^c^	*− 116,441*

^a^Model abbreviations follow LP.*x.y*.R*z*, where LP stands for Legendre polynomial, *x* is the polynomial order for the additive genetic, *y* is the polynomial order for the permanent environmental effects and *z* is the residual variance structure defined as ho: homogeneous; he: heterogeneous residual variance based on 5 categories of staple length; fy: heterogeneous residual variance for each flock-year)

^b^Selection criteria; BIC = Bayesian information criterion, PAL = penalizing adaptive the likelihood

^c^Values in italics indicate the optimal model for each criterion

### Variance components, heritability and genetic correlations

**Fig. 2:**
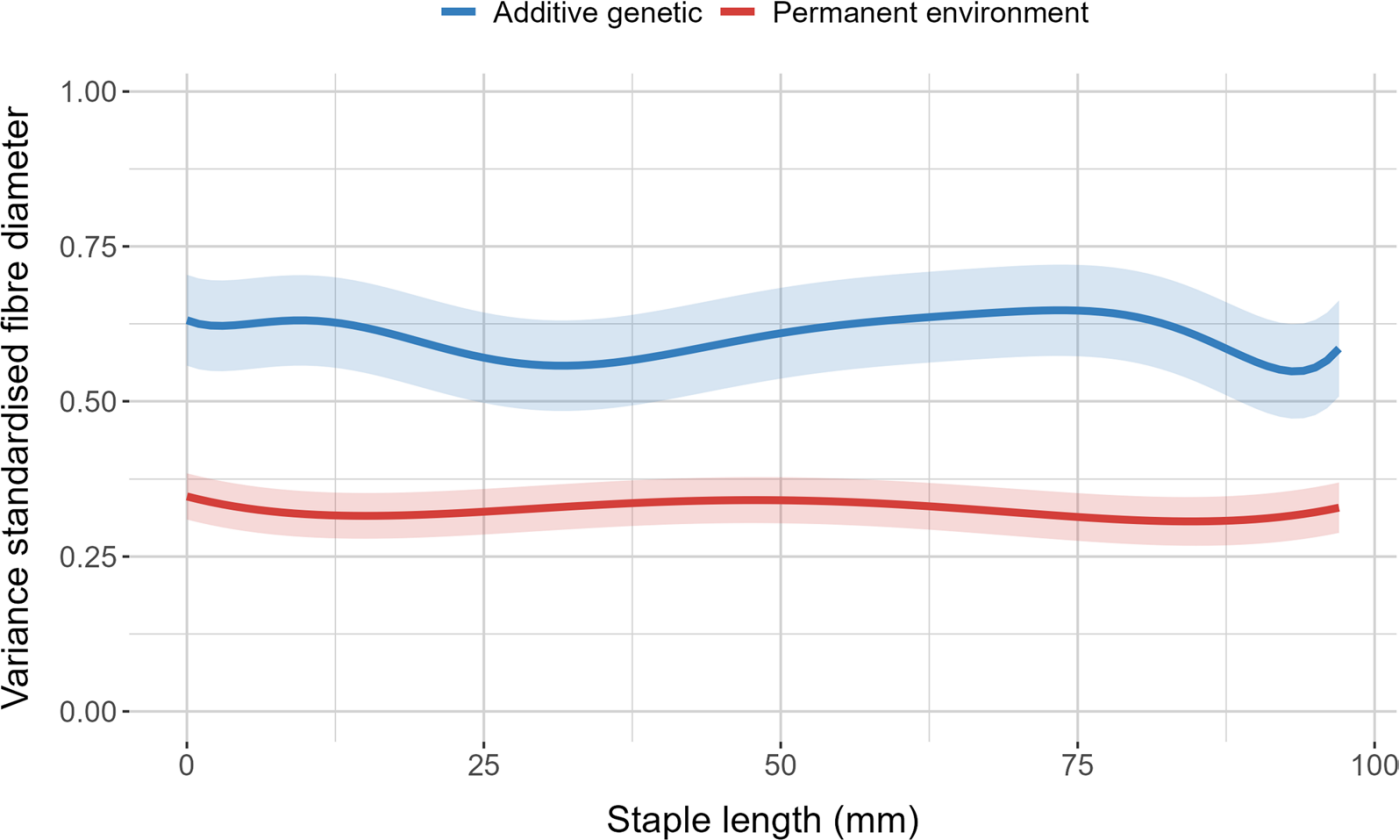
Additive genetic and permanent environmental variance of standardised fibre diameter according to staple length (mm). Shading indicates the approximate standard error

**Fig. 3:**
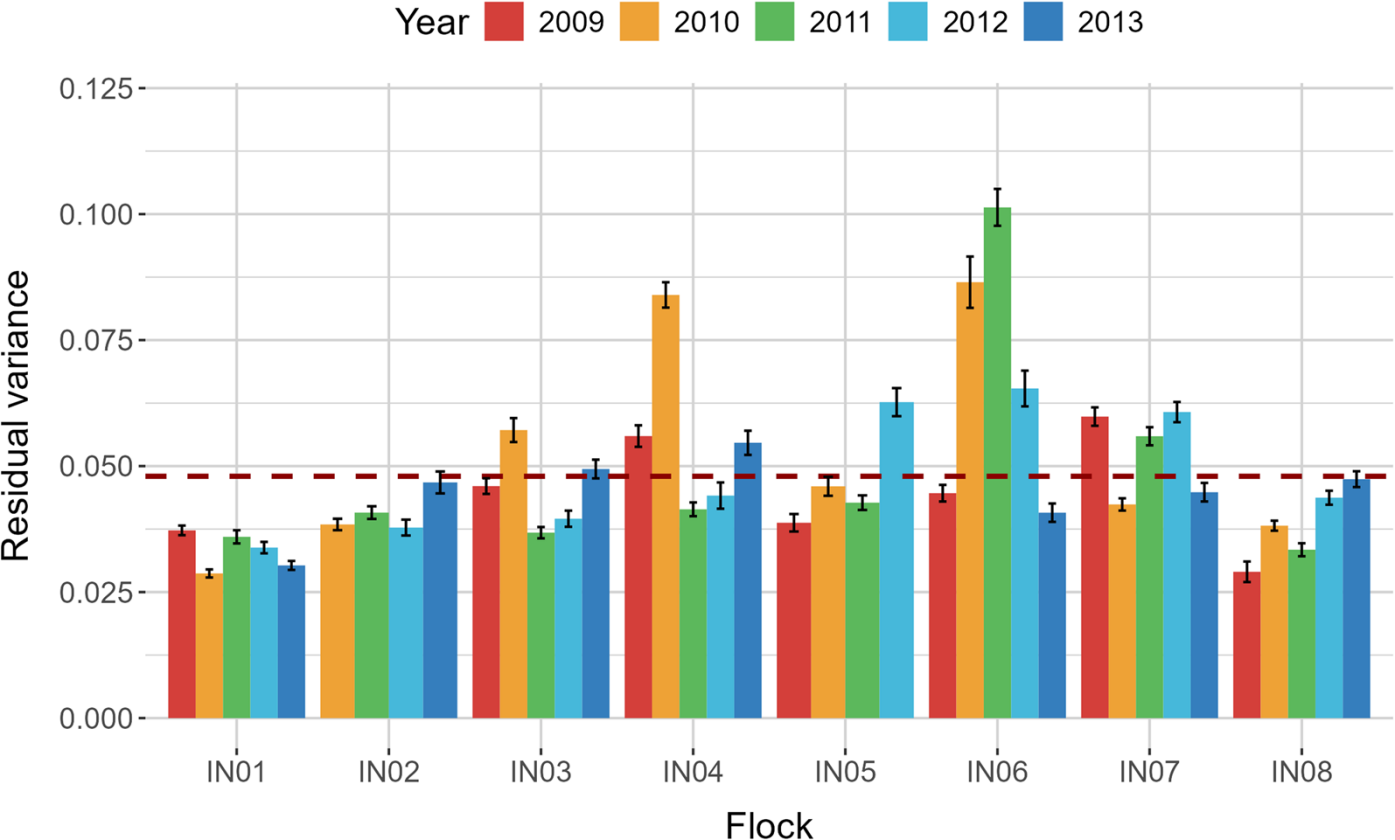
Residual variance estimates for each flock-year using a random regression model of standardised fibre diameter over the length of the staple. The broken horizontal line indicates the mean residual variance. Error bars indicate the approximate standard error of the residual variance for each flock-year

**Fig. 4:**
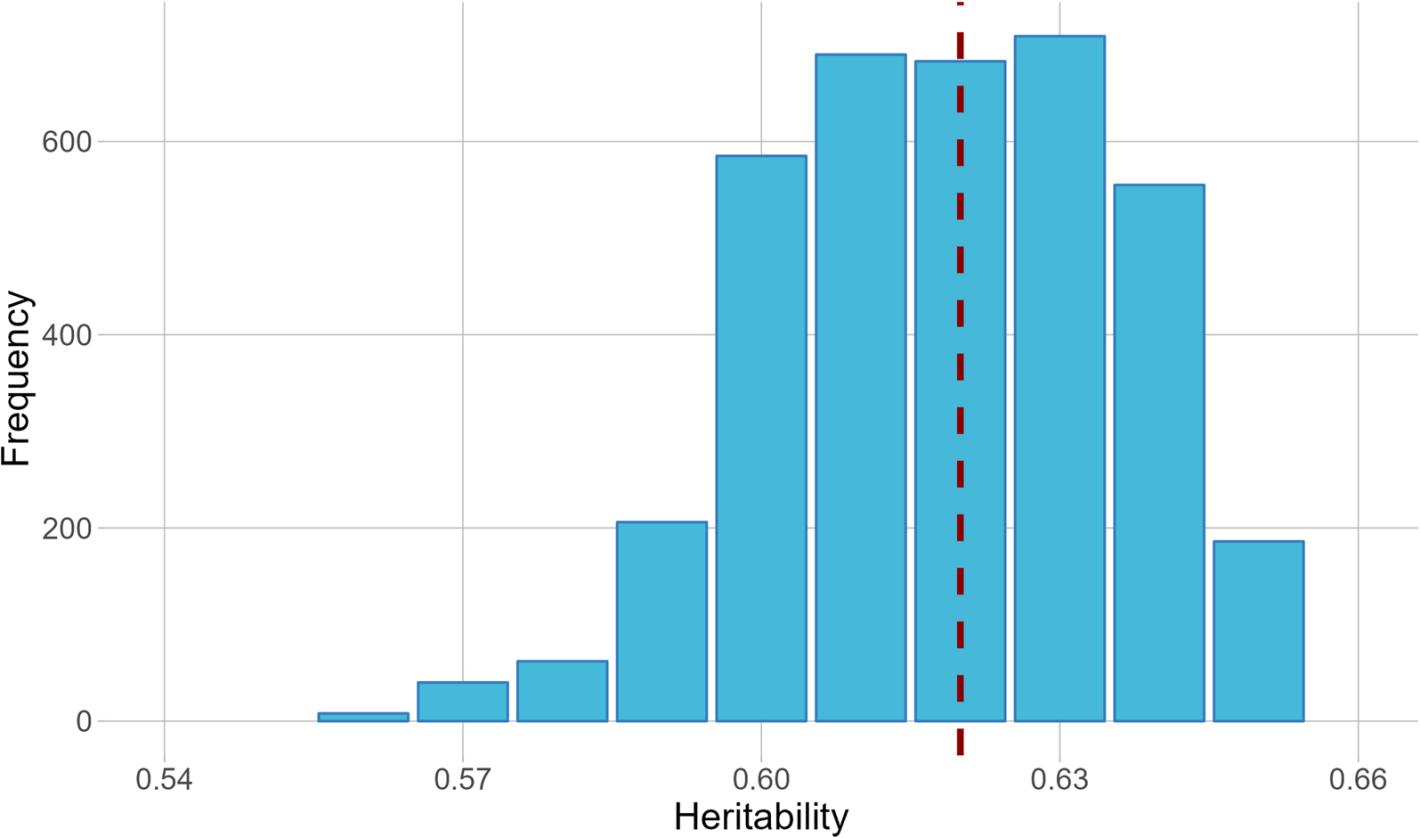
Distribution of heritability estimates of standardised fibre diameter along the length of the staple combined for all flock-years

The additive genetic and permanent environmental variances of fibre diameter estimated along the staple length are shown in Fig. 2. The highest additive genetic variance was at ~ 75 mm (0.65) while the lowest values were estimated at 30 mm and 90 mm (0.55), the approximate standard errors indicated that these differences were not statistically significantly different. The permanent environmental variance was consistent along the staple length ranging between 0.30 to 0.35. The residual variances defined for each flock-year were variable (Fig. 3) and were small compared to the additive and permanent environmental variance. The highest residual variance was observed for IN06_2011 with a residual variance of 0.10. The lowest residual variances were observed for IN01_2010 and IN08_2009, with a residual variance of 0.03. Together the heritability estimates of standardised fibre diameter measured across each of the flock-years are summarised in Fig. 4. The heritability ranged between 0.56 to 0.65 with a mean heritability of 0.62. The heritability estimates of standardised fibre diameter at each staple length increment for the two flocks-years with the lowest and highest residual variance are shown in Additional file 6: Figure S4. The heritabilities of standardised fibre diameter at each increment resembled a similar pattern to the additive genetic effects, showing higher heritabilities in the middle portion of the staple and lower at the extremities.

**Fig. 5:**
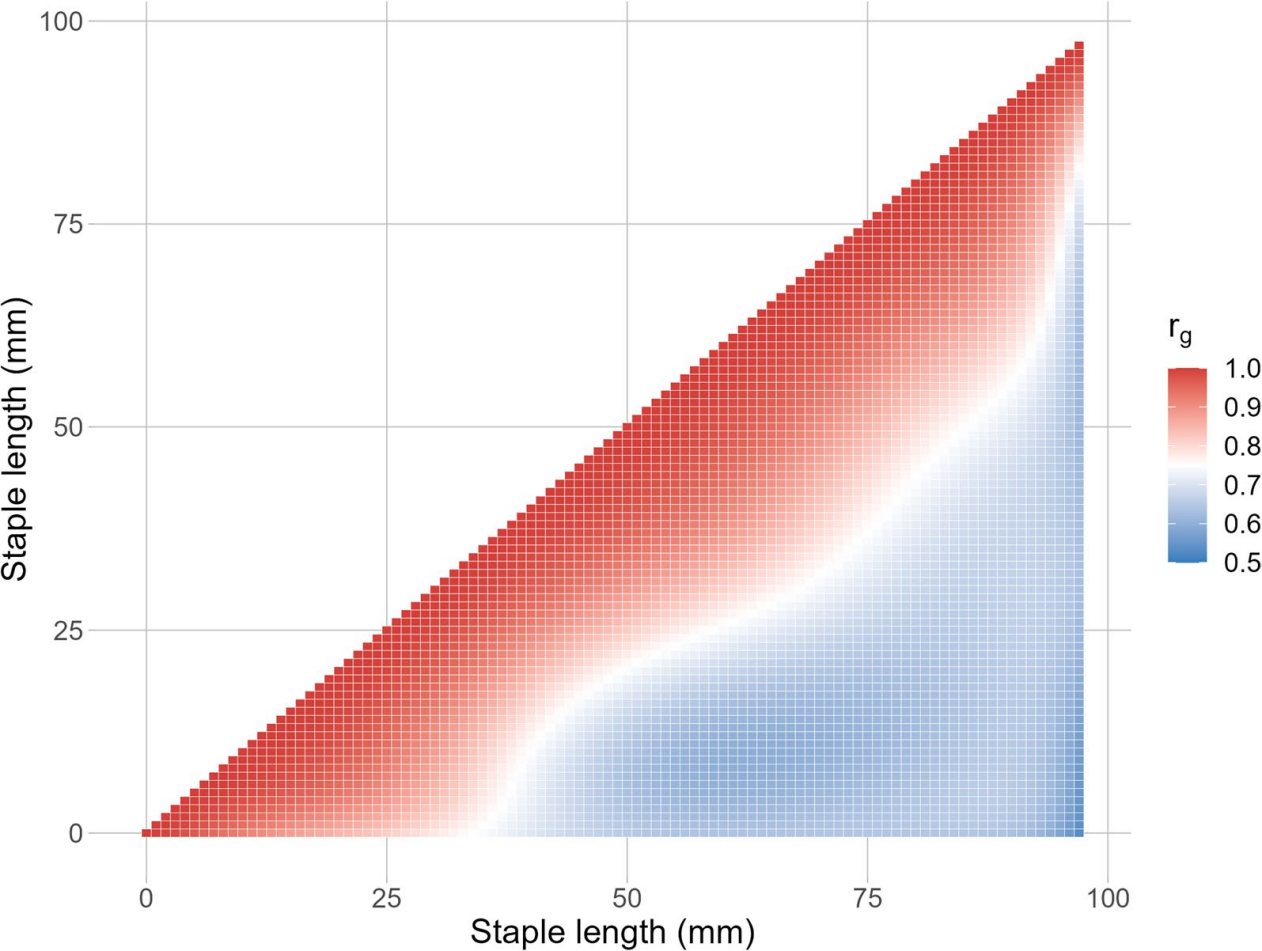
Estimated genetic correlations (r_g_) for standardised fibre diameter at different increments of staple length (mm)

The genetic correlations of standardised fibre diameter estimated at each increment along the staple length ranged between 0.53 and 1.00 (Fig. 5). In particular, the genetic correlations between sequential fibre diameter measurements were at or close to unity, however, the correlation between early (0–25 mm) and later measurements (75–97 mm) was only moderate ranging between 0.53 and 0.58. Despite the smaller genetic correlations among more distant fibre diameter records, all estimates were positive. This indicates that the selection of fibre diameter at any given staple length will be accompanied by positive responses to all other points along the staple length.

**Fig. 6:**
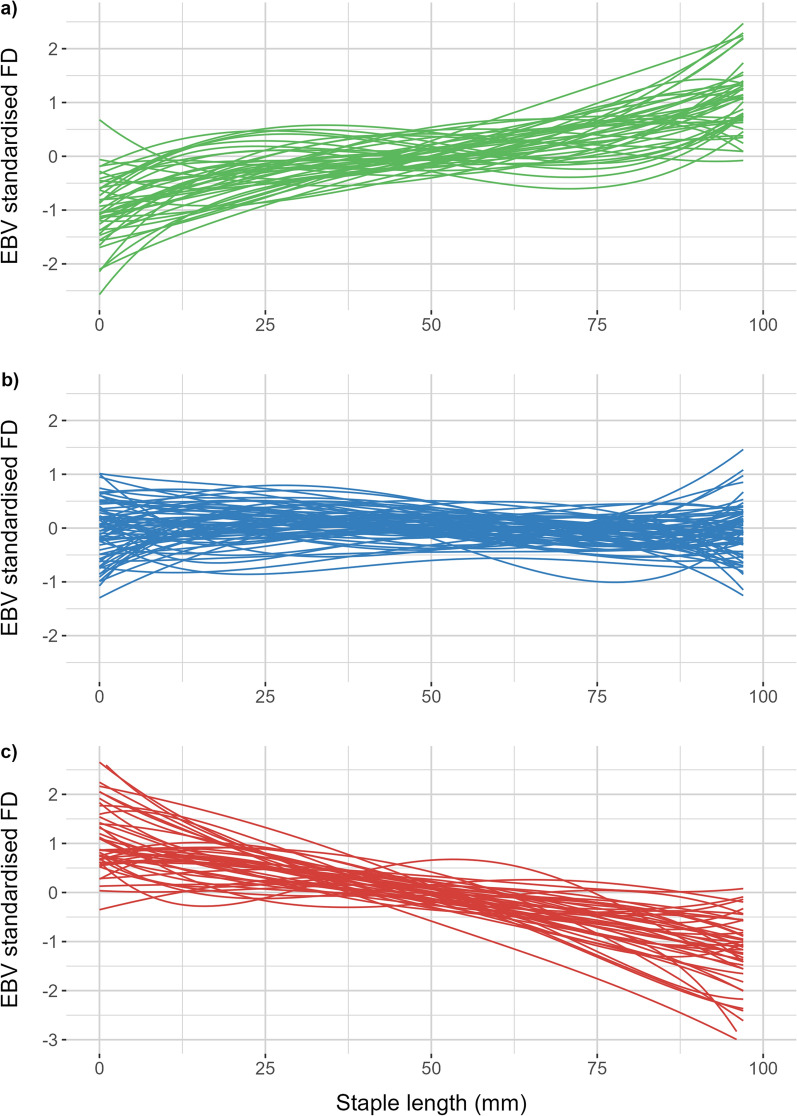
Change in sire estimated breeding values (EBV) for standardised fibre diameter (FD) along the length of the wool staple. Each line represents one sire categorised into one of three groups (**a**) sires with EBVs that increase with staple length (n = 44); (**b**) Sires with EBVs that tend to remain approximately constant along the staple within − 1 and + 1 genetic standard deviations (n = 89); or (**c**) Sires with EBVs that decrease along the staple (n = 43)

Figure 6 shows the change in sire EBV for standardised fibre diameter between the beginning and end of the staple length. Three groups of animals were identified based on visual inspection of the change in EBV over the staple length. In the first group (Fig. 6a) sires were identified as having EBV for standardised fibre diameter which overall increased with increasing staple length (change greater than 1 genetic standard deviation), while the third group (Fig. 6c) showed the opposite tendency with decreasing EBV with increasing staple length (change greater than 1 genetic standard deviation). The second group included animals that showed intermediate values near or close to zero but within − 1 to + 1 genetic standard deviations (Fig. 6b). The number of progeny per sire and the number of flocks in which each sire was represented did not appear to correlate with the group into which the sire was categorised as shown in Fig. 6. The majority of sires were within the second group (50.5%) whereas the remaining sires were categorised approximately equally in the first and third groups (25% and 24.5%, respectively). Animals with steeper curves were more sensitive to their environment and consequently less resilient, compared to sires with more uniform curves.

The Spearman rank correlation between fibre diameter EBVs of sires estimated at five length increments showed a re-ranking of genotypes along the staple length (Table 2). Fibre diameter EBVs for increments in close proximity at either end of the profiles (0–25 mm and 75–95 mm) showed high EBV rank correlation, while fibre diameter measures that were further apart tended to produce moderate negative correlations (− 0.53 to − 0.66) suggesting re-ranking. This, combined with the results of Figs. 5 and 6 demonstrates that there is genetic variation in the sensitivity of fibre diameter along the staple length.

**Table 2:** Spearman correlations between sire EBV for standardised fibre diameter estimated at 5 staple lengths

	Spearman correlation (95% CI)^a^					
Staple length (mm)	0		25	50	75	95
0			0.65 (0.54 to 0.73)	− 0.17 (− 0.32 to − 0.02)	− 0.66 (− 0.74 to − 0.57)	− 0.53 (− 0.68 to − 0.47)
25				0.20 (0.05 to 0.35)	− 0.64 (− 0.73 to − 0.54)	− 0.59 (− 0.69 to − 0.48)
50					0.29 (0.14 to 0.43)	0.05 (− 0.10 to 0.21)
75						0.79 (0.72 to 0.84)
95						

^a^95% confidence interval (Cl) in parenthesis

**Fig. 7:**
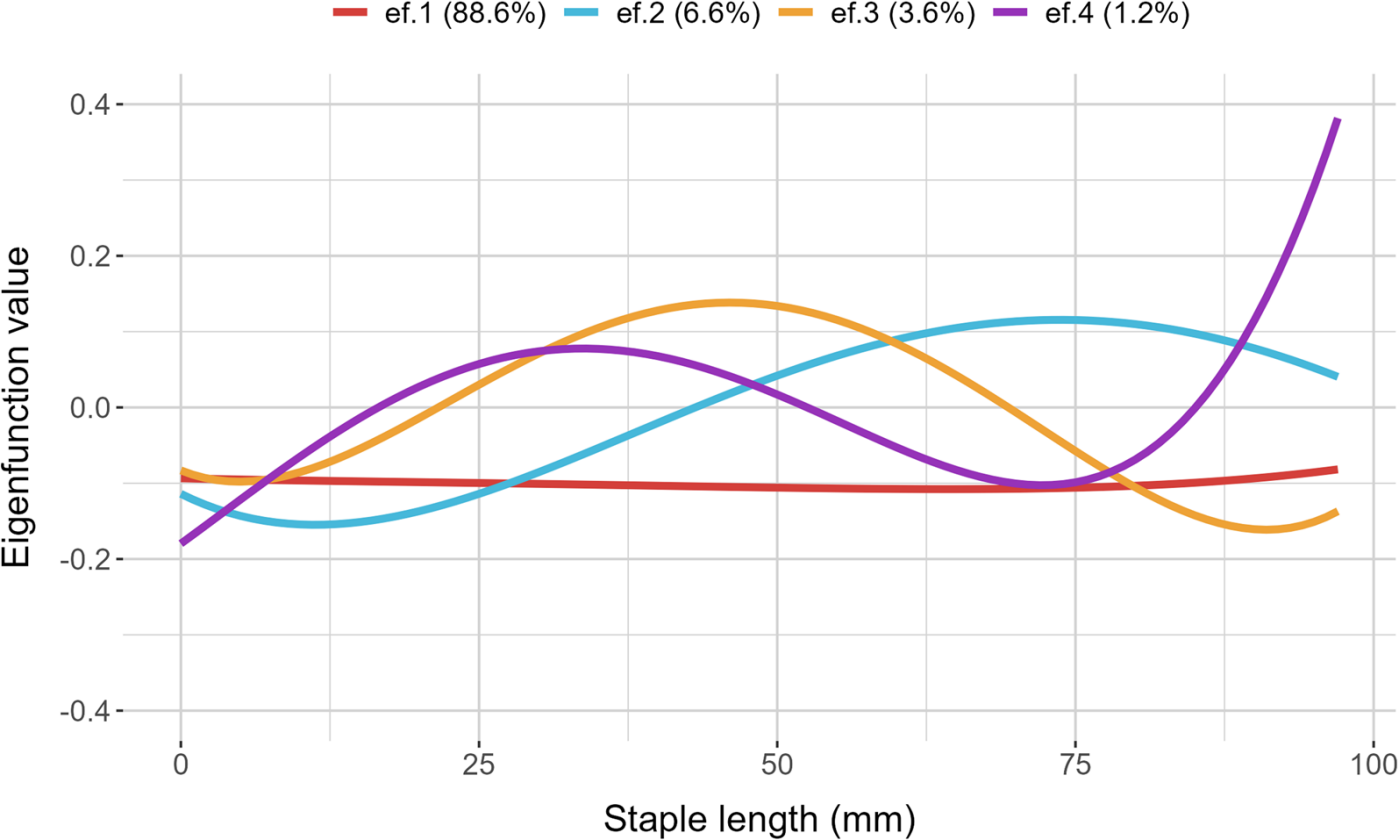
The first (ef.1), second (ef.2), third (ef.3) and fourth (ef.4) eigenfunction values for the (co)variance matrix of the additive genetic regression coefficients of standardised fibre diameter at different increments along the staple length. The proportional eigenvalues are shown in parentheses

The eigenfunctions corresponding to the eigenvectors for the (co)variance matrix for the additive genetic regression coefficients are presented in Fig. 7. The first eigenfunction was estimated to account for 88.6% of the total genetic variation and was constant over the staple length. The second, third and fourth eigenfunctions accounted for 6.6%, 3.6% and 1.2% of the variation, respectively. The second and fourth eigenfunctions showed a sigmoidal tendency while the third eigenfunction had a concave shape over the length of the staple. Selection on the first eigenfunction would permit changing the fibre diameter at all staple lengths along the fibre in the same direction. Comparatively, selection on the second eigenfunction would be expected to decrease fibre diameter at the beginning of staple and increase fibre diameter at higher staple length increments. Selection on the third and fourth eigenfunctions would be expected to produce the opposite results to the second eigenfunction, albeit these two eigenfunctions make up a much smaller proportion of the genetic variation.

## Discussion

Quantifying the resilience of extensively raised animals to environmental disturbances remains an ongoing challenge in animal breeding. In this study, the effects of environmental disturbances on the sheep were assessed indirectly through changes in fibre diameter along the staple length. Variation in fibre diameter along the length of the wool staple was considered due to its known association with environmental disturbances such as nutrition, management, pregnancy, climatic conditions and parasitic diseases [10, 32, 33]. The application of random regression models to this data was used to account for the average fibre diameter trajectory of each flock-year groups plus a set of random regression coefficients that quantify the individual deviation. An optimal fit of the random regression model was required to infer the resilience of genotypes to the environment.

### Model fit

The selection of the most appropriate function for a random regression model is crucial to capturing the shape of fixed and random regressions and making correct inferences about the change in fibre diameter over the staple length. Here the optimal model (LP.4.2Rfy) applied Legendre polynomials of orders five, four and two, to the fixed, additive and permanent environmental effects respectively, while considering the residual variance to be heterogenous for each flock-year. The superior fit of this model compared to the other models tested was attributable to the higher order Legendre polynomial of the additive effect and the more accurate modelling of the residual variance. By allowing the residual variances to be estimated separately for different environments within the model, it was more flexible and realistic in capturing the specific variations in each flock-year.

The Legendre polynomials used in this study were typically higher order for both fixed and additive genetic effects compared to other longitudinal traits such as growth [15]. This was warranted given the complexity of some of the flock-year curves. Fibre diameter curves can take complex shapes because, firstly, there is no biological process that underpins the shape of the curve, unlike traits such as lactation, growth or egg production. Secondly, the fibre diameter curve is dictated primarily by the overall quality of the environment and where the sampling events occur (determining the two ends of the fibre) [11, 34]. However, fitting Legendre polynomials to higher orders can lead to issues including overfitting, unrealistic variance estimates at the extremes of the trajectory and higher computational complexity [35, 36]. Future work on random regression modelling of fibre diameter could investigate the use of different covariance functions such as cubic smoothing or B-splines. The use of splines with different numbers and locations of knots may offer higher flexibility in modelling the fixed regression which could then lead to improvements in the estimation of individual deviation from the fixed regression [36]. Despite these considerations, improvements in the overall model fit and parameter estimates using splines would be expected to be minimal considering the magnitude of the additive genetic variance of fibre diameter.

### Variance components and heritabilities

The additive genetic variance showed two points of inflection located at the middle and end of the staple. Specifically, there was a relatively sharp decrease toward the end of the trajectory beyond 75 mm, although not significant (Fig. 2). Similar behaviour of covariance function estimates have been shown at the ends of lactation and growth trajectories where fewer data points are located [37, 38]. While the staple length in this study was standardised, Fischer and van der Werf [39] showed that end point measures can still be affected when the data is evenly distributed, particularly with the use of higher order Legendre polynomials. Departure from the use of polynomials into other covariance functions such as splines, may be able to alleviate this issue. Despite these concerns, the overall variance partitioning is similar to that shown by studies using random regression models to estimate variance parameters of fibre diameter across age classes in sheep and alpacas [40, 41].

Together the heritability of standardised fibre diameter along the staple length ranged between 0.56 and 0.65 across different flock-years. These estimates align with the typically high heritability estimates of mean fibre diameter which are reported to range between 0.54 and 0.67 in Australian Merinos [42, 43]. Pollott and Greeff [44] however, found lower heritability estimates of mean fibre diameter (0.40–0.50) when estimated from a reaction norm where environments were defined based on contemporary group means. These differences may be attributable to the larger population size or could reflect the diversity of genetic lines captured within the flocks (industry and research flocks) analysed by Pollott and Greeff [44], compared to the current study. Overall, the variance components and heritabilities estimated here indicate variation in the performance of fibre diameter along the wool staple exists, and support earlier studies showing that the variability of fibre diameter has a heritable genetic component [6, 13].

### Resilience and the potential to alter fibre diameter curves

A re-ranking of genotypes was observed along the staple length which shows that there was genetic variation in the sensitivity of fibre diameter performance to different growing environments. In this case, the length of the fibres within a staple is expected to represent the animal’s interaction with different environments and disturbances experienced throughout the wool growth period. The lower genetic correlation between standardised fibre diameter measured at the extremes of the staple length further suggests that re-ranking of animals is occurring. While there are few similar estimates in the literature, Dominik et al. [45] showed that the genetic correlation between mean fibre diameter measured across environments was moderate with a value of 0.77. This re-ranking may infer a lack of adaption of a particular genotype to a specific condition, with the slope of the curves said to represent the tolerance (or resilience towards) environmental conditions [2]. Sires with steeper curves (Fig. 6a, c) are said to be more affected by environmental disturbances compared to sires with flatter curves closer to zero. This may be important when it comes to choosing animals that are resilient to the stresses of the production system. Berghof et al. [2] however, noted that this method only quantified resilience towards environmental disturbance at the macro-environmental level which could be a potential limitation of this current method. It may be expected that information about animal resilience towards short term disturbances could be gained from analysing fibre diameter variation using other methods. For example, recent studies have analysed the genetic variation in the deviations of longitudinal traits as an indicator of resilience in pigs, poultry and dairy cattle [46–48]. Trait definitions rely on the prediction of an overall or average curve with the individual deviation from the overall curve calculated using metrics such as skewness, autocorrelation and the log-transformed variance of the deviation. These characteristics describe more closely the response and recovery from environmental disturbances on a shorter time scale rather than the overall trend. Similar methods could be used on fibre diameter profile data and could complement the current findings.

The structure of the eigenfunction and the size of the associated eigenvalues indicate the extent to which the fibre diameter profile curves can be altered through selection. The consistency of the first eigenfunction and the size of the associated eigenvalue (88.6%) implies that the majority of the variance is explained by genetic factors that are constant for all measurements made along the staple. This result is consistent with other longitudinal traits and studies of genotype by environment studies (e.g., Bermejo et al., [49] and Soumri et al. [50]). The associated EBV for these eigenvectors corresponds to the average potential of the animal across the staple, in this case, the overall mean fibre diameter. In comparison, selection on the remaining eigenfunctions would allow changing the shape of the variation in fibre diameter along the length of the staple. Specifically, selection on the second eigenfunction would permit an increase in fibre diameter in the last three-quarters of the staple length. This has particular relevance to resilience given that steep negative slopes are the most undesirable and could be considered to indicate reduced tolerance to environmental disturbances [51, 52]. In dairy cattle, selection indexes are often created from the first three eigenfunctions to balance milk production and the shape of the lactation curve (persistency) [53, 54]. Similarly, a selection index could be created to permit selection on the mean fibre diameter and the variability of fibre diameter (environmental sensitivity). However, as the first eigenvector explained the largest proportion (> 80%) of the genetic variation, strategies to alter the shape of the fibre diameter profile would require greater selection pressure on the relevant eigenfunction.

The possibility of selecting for a uniform fibre diameter profile raises questions as to whether this is a desirable characteristic to breed for from a resilience perspective. This study was conducted primarily under the assumption that fibre diameter variability over the length of the staple is the result of challenges experienced by the animal between shearing events. This was informed by experimental and field experiments demonstrating the link between factors such as nutrition, climate, diseases, parasites and management stress with periods of declining fibre diameter [10, 11, 33, 55]. Since fibre diameter variation is believed to be caused by acute perturbation it could be anticipated that the incidence and severity of this variation would be positively correlated phenotypically and genetically with existing health, welfare and production traits of sheep. Selecting sires that have more uniform breeding values for fibre diameter across the wool staple could also have a positive economic impact on wool quality. Phenotypically, a wool staple that has high variability in fibre diameter along its length will also have reduced staple strength (force required to break an individual staple when extended). Staple strength is regarded as the second most important wool quality characteristic after fibre diameter and is a key determinant of the processing performance of apparel wool. Selection against a convex shaped profile would be expected to benefit staple strength given convex profiles are subject to greater load stress upon extension [56]. Together, these correlated responses need to be confirmed before applying selection on this novel trait in breeding programs. Such work is also anticipated to provide further guidance as to what should be considered the ‘ideal’ phenotype for this longitudinal trait.

Evaluating changes in fibre diameter along a wool staple at the genotypic level is an emerging space for resilience research. It is therefore unclear if the results of the current study and those previously, which were based on simple trait definitions [6] adequately capture variation in fibre diameter to be used as a measure of resilience. While random regression models take account of the continuity of the fibre diameter trajectory across the fibre staple, one limitation is they do not explicitly produce a trait that could be introduced into a breeding program. Breeding for a certain shape of profile would require the construction of an index based on the eigenfunctions, which can often be cumbersome to interpret. Other studies have created “pseudo-phenotypes” from the random regression model based on the slope and/or curve components to explicitly describe the sensitivity of the trait [57, 58] or converted multiple EBVs into a single estimate, as in the case of persistency phenotypes of dairy cattle (e.g., Cobuci et al. [59]). Further work on the use of random regression models with fibre diameter could incorporate some of these methods to create a trait that is more suited for inclusion in breeding programs. The possibility also remains to investigate the genetic basis of the variation itself. For instance, there may be value in determining if certain genetic variants are associated with a shape parameter that describes different fibre diameter curves.

Further studies on fibre diameter profiles should also consider how to standardise the length of the wool staple to a common scale, as this may have some implications on the interpretation. Here the fibre diameter profiles were standardised as a proportion of their maximum length under the assumption that fibre growth throughout the growing period was equal. This approach was taken in the absence of additional information that would enable the determination of the rate of fibre elongation throughout the year. The assumption of equal growth using standardisation techniques has been used to approximate different growing environments well in other works [11, 33]. However, Hynd [60] and Schlink et al. [61], showed that the rate of fibre elongation varied between 268–506 μm/day and 374–434 μm/day, respectively, due to nutritional treatments resulting in differences between animals in their overall staple length. This means that the distance between fibre diameter measurements likely differs throughout the year. Further work should investigate techniques for standardisation of fibre diameter profiles to known timed events to overcome this assumption.

## Conclusions

This study demonstrated genetic variation in standardised fibre diameter measured at different increments along the length of the wool staple using a random regression model. This result indicates the potential to select animals based on the shape of the fibre diameter profile curve which could be used to reduce the sensitivity of sheep towards environmental disturbances. Further work is required to validate these results against existing measures of health, welfare and productivity and to refine the methodology for assessing resilience towards environmental disturbances. Fibre diameter profiles however remain a promising area of research to investigate the resilience in extensively raised livestock, for which, appropriate metrics are currently lacking.

## Supplementary Information

Additional file 1: Figure S1. The distribution of the number of fibre diameter records per animaland the distribution of the standardised fibre diameter records at different staple lengths.

Additional file 2: Table S1. Structure of the dataset used to evaluate standardised fibre diameter along the wool staple at each of the eight Information Nucleus Flock sites.

Additional file 3: Figure S2. Example of the standardised fibre diameter values plotted along the wool staple for Information Nucleus Flocks 1 to 8 during the 2011 year of recording. The red line indicates the average curve estimates for each flock year combination.

Additional file 4: Table S2. Bayesian Information Criteria, F statistics and partial F-test results from fixed regression of models containing Legendre polynomials of orders 1 to 6.

Additional file 5: Figure S3. Residual versus fitted plots of standardised fibre diameter from A) MOD1 and B) MOD5 which test the order of Legendre polynomial applied to the fixed regression curve. In a well-behaved linear regression model, the residuals should exhibit constant varianceacross all levels of the fitted values. The green line represents the locally weighted scatterplot smoothing linewhich helps visualise whether the spread of the residuals remains roughly constant as the fitted values change. If the spread of the residuals widens or narrows systemically as the fitted values increase or decrease it suggests heteroscedasticity which violates the assumption of constant variance.

Additional file 6: Figure S4. Heritability estimates of standardised fibre diameter measured along the wool staple for the flock years with the lowestand highestresidual variance estimates. The approximate standard error of the heritability estimates ranged between 0.089 and 0.095 for each flock-year.

## Data Availability

The data used in this study was not deposited in an official repository. The data are owned by Meat and Livestock Australia. Access to the data can be negotiated upon request.

## References

[1] Colditz IG, Hine BC. Resilience in farm animals: biology, management, breeding and implications for animal welfare. Anim Prod Sci. 2016;56:1961.

[2] Berghof TVL, Poppe M, Mulder HA. Opportunities to improve resilience in animal breeding programs. Front Genet. 2019;9:692. 30693014 10.3389/fgene.2018.00692 PMC6339870

[3] Friggens N, Adriaens I, Boré R, Cozzi G, Jurquet J, Kamphuis C, Leiber F, Lora I, Sakowski T, Statham J, De Haas Y. Resilience: reference measures based on longer-term consequences are needed to unlock the potential of precision livestock farming technologies for quantifying this trait. PCI. 2022;2: e38.

[4] Colditz IG, Smith EG, Ingham AB, Dominik S. Indicators of functional integrity in production animals. Anim Prod Sci. 2023;63:825–43.

[5] Dominik S, Swan A. Resilience, tolerance, robustness and genotype × environment interaction in Merino sheep breeding. In: Hermesch S, Dominik S, editors. Breeding Focus 2014—Improving resilience. Animal Genetics and Breeding Unit; 2014. p.115–27.

[6] Smith EG, Walkom SF, Clark SA. Exploring genetic variation in potential indicators of resilience in sheep using fibre diameter measured along the wool staple. Animal. 2024;18:101065. 38237476 10.1016/j.animal.2023.101065

[7] Masters DG, Mata G, Liu SM, Peterson AD. Influence of liveweight, liveweight change, and diet on wool growth, staple strength, and fibre diameter in young sheep. Aust J Agric Res. 1998;49:269–77.

[8] Rogers GE. Biology of the wool follicle: an excursion into a unique tissue interaction system waiting to be re-discovered. Exp dermatol. 2006;15:931–49. 17083360 10.1111/j.1600-0625.2006.00512.x

[9] Thompson A, Hynd P. Wool growth and fibre diameter changes in young Merino sheep genetically different in staple strength and fed different levels of nutrition. Aust J Agric Res. 1998;49:889–98.

[10] Walkden-Brown S, Daly B, Colditz I, Crook B. Role of anorexia in mediating effects of blowfly strike on wool. Asian-Australas J Anim Sci. 2000;13:76–9.

[11] Gonzalez EB, Sacchero DM, Easdale MH. Environmental influence on Merino sheep wool quality through the lens of seasonal variations in fibre diameter. J Arid Environ. 2020;181: 104248.

[12] Schlink A, Mata G, Lea J, Ritchie A. Seasonal variation in fibre diameter and length in wool of grazing Merino sheep with low or high staple strength. Aust J Exp Agric. 1999;39:507–17.

[13] Preston J, Hatcher S. Genetic estimates for along and across fibre diameter variation and its use to improve staple strength in Merino sheep. Proc Assoc Advmt Anim Breed Genet. 2013;20:106–9.

[14] Schaeffer LR. Application of random regression models in animal breeding. Livest Prod Sci. 2004;86:35–45.

[15] Oliveira H, Brito L, Lourenco D, Silva F, Jamrozik J, Schaeffer L, Schenkel FS. Invited review: advances and applications of random regression models: from quantitative genetics to genomics. J Dairy Sci. 2019;102:7664–83. 31255270 10.3168/jds.2019-16265

[16] Fogarty N, Banks R, van Der Werf J, Ball A, Gibson J. The information nucleus—a new concept to enhance sheep industry genetic improvement. Proc Assoc Advmt Anim Breed Genet. 2007;17:29–32.

[17] van der Werf J, Kinghorn B, Banks R. Design and role of an information nucleus in sheep breeding programs. Anim Prod Sci. 2010;50:998–1003.

[18] Geenty K, Brien F, Hinch G, Dobos R, Refshauge G, McCaskill M, Ball AJ, Behrendt R, Gore KP, Savage DB, Harden S. Reproductive performance in the Sheep CRC Information Nucleus using artificial insemination across different sheep-production environments in southern Australia. Anim Prod Sci. 2014;54:715–26.

[19] Scobie DR, Grosvenor AJ, Bray AR, Tandon SK, Meade WJ, Cooper AMB. A review of wool fibre variation across the body of sheep and the effects on wool processing. Small Rumin Res. 2015;133:43–53.

[20] Baxter BP, Brims MA, Taylor TB. Description and performance of the optical fibre diameter analyser (OFDA). J Text Inst. 1992;83:507–26.

[21] Brims M, Peterson A, Gherardi S. Introducing the OFDA2000—for rapid measurement of diameter profile on greasy wool staples. International Wool Textile Organisation: Report No: RWG 04, 1999. p. 1–8.

[22] Swan AA, Brown DJ, van der Werf JH. Genetic variation within and between subpopulations of the Australian Merino breed. Anim Prod Sci. 2015;56:87–94.

[23] D’Arcy JB. Sheep management and wool technology. Kensington: New South Walse University Press LTD; 1990.

[24] Butler D, Cullis B, Gilmour A, Gogel B, Thompson R. ASReml-R reference manual. Version 4.1. 0.130. Hemel Hempstead, UK: VSN International Ltd; 2020.

[25] Duncan DB. Multiple range and multiple F tests. Biometrics. 1955;11:1–42.

[26] Schwarz G. Estimating the dimension of a model. Ann Stat. 1978;6:461–4.

[27] R Core Teams. R: a language and environment for statistical computing. Vienna : R Foundation for Statistical Computing; 2023.

[28] Stoica P, Babu P. Model order estimation via penalizing adaptively the likelihood (PAL). Signal Process. 2013;93:2865–71.

[29] Corrales JD, Munilla S, Cantet RJC. Polynomial order selection in random regression models via penalizing adaptively the likelihood. J Anim Breed Genet. 2015;132:281–8. 25622858 10.1111/jbg.12130

[30] Fischer TM, Gilmour AR, van der Werf JH. Computing approximate standard errors for genetic parameters derived from random regression models fitted by average information REML. Genet Sel Evol. 2004;36:363–9. 15107271 10.1186/1297-9686-36-3-363 PMC2697206

[31] Kirkpatrick M, Lofsvold D, Bulmer M. Analysis of the inheritance, selection and evolution of growth trajectories. Genetics. 1990;124:979–93. 2323560 10.1093/genetics/124.4.979 PMC1203988

[32] James P, Moon R, Brown D. Seasonal dynamics and variation among sheep in densities of the sheep biting louse, Bovicola ovis. Int J Parasitol. 1998;28:283–92. 9512991 10.1016/s0020-7519(97)00188-4

[33] Whelan MB, Geenty K, Cottle D, Lamb DT, Donald G. The relationship between a satellite derived vegetation index and wool fibre diameter profiles. In: Proceedings of 10th World Conference on Animal Production: 23–28 November 2008; Cape Town. 2008.

[34] Smith J, Purvis I, Lee G. Fibre diameter profiles-potential applications for improving fine-wool quality. Wool meets meat: tools for a modern sheep enterprise. In: Proceedings of the 2006 Australian Sheep Industry Cooperative Research Centre Conference: 22–23 February 2006; Orange. 2006.

[35] Meyer K. Random regression analyses using B-splines to model growth of Australian Angus cattle. Genet Sel Evol. 2005;37:473. 16093011 10.1186/1297-9686-37-6-473 PMC2697221

[36] Misztal I. Properties of random regression models using linear splines. J Anim Breed Genet. 2006;123:74–80. 16533360 10.1111/j.1439-0388.2006.00582.x

[37] van der Werf J, Goddard M, Meyer K. The use of covariance functions and random regressions for genetic evaluation of milk production based on test day records. J Dairy Sci. 1998;81:3300–8. 9891276 10.3168/jds.S0022-0302(98)75895-3

[38] Fischer TM, Van der Werf JHJ, Banks RG, Ball AJ. Description of lamb growth using random regression on field data. Livest Prod Sci. 2004;89:175–85.

[39] Fischer T, van der Werf J. Effect of data structure on the estimation of genetic parameters using random regression. In: Proceedings of the 7th World Congress on Genetics Applied to Livestock Production: August 2002; Montpellier. 2002.

[40] Fozi MA, van der Werf J, Swan A. Modelling genetic covariance structure across ages of mean fibre diameter in sheep using multivariate and random regression analysis. Anim Prod Sci. 2012;52:1019–26.

[41] Cruz A, Menéndez-Buxadera A, Gutiérrez G, Morante R, Burgos A, Gutiérrez JP. Genetic (co) variance across age of fiber diameter and standard deviation in Huacaya alpacas, estimated by repeatability, multi-trait and random regression models. Livest Sci. 2020;231: 103863.

[42] Safari E, Fogarty NM, Gilmour AR. A review of genetic parameter estimates for wool, growth, meat and reproduction traits in sheep. Livest Prod Sci. 2005;92:271–89.

[43] Safari E, Fogarty NM, Gilmour AR, Atkins KD, Mortimer SI, Swan AA, Brien FD, Greeff JC, van der Werf JH. Genetic correlations among and between wool, growth and reproduction traits in Merino sheep. J Anim Breed Genet. 2007;124:65–72. 17488356 10.1111/j.1439-0388.2007.00641.x

[44] Pollott G, Greeff J. Genotype x environment interactions and genetic parameters for fecal egg count and production traits of Merino sheep. J Anim Sci. 2004;82:2840–51. 15484934 10.2527/2004.82102840x

[45] Dominik S, Crook B, Kinghorn B. Genotype x management interaction on wool production traits and body weight in Western Australian merino sheep. Proc Assoc Advmt Anim Breed Genet. 1999;13:98–101.

[46] Elgersma GG, de Jong G, van der Linde R, Mulder HA. Fluctuations in milk yield are heritable and can be used as a resilience indicator to breed healthy cows. J Dairy Sci. 2018;101:1240–50. 29174159 10.3168/jds.2017-13270

[47] Gorssen W, Winters C, Meyermans R, Chapard L, Hooyberghs K, Janssens S, et al. A promising resilience parameter for breeding: the use of weight and feed trajectories in growing pigs. J Anim Sci Biotechnol. 2023;14:101. 37525252 10.1186/s40104-023-00901-9 PMC10391771

[48] Bedere N, Berghof TVL, Peeters K, Pinard-van der Laan MH, Visscher J, David I, et al. Using egg production longitudinal recording to study the genetic background of resilience in purebred and crossbred laying hens. Genet Sel Evol. 2022;54:26. 35439920 10.1186/s12711-022-00716-8 PMC9020098

[49] Bermejo JL, Roehe R, Schulze V, Rave G, Looft H, Kalm E. Random regression to model genetically the longitudinal data of daily feed intake in growing pigs. Livest Prod Sci. 2003;82:189–99.

[50] Soumri N, Carabaño MJ, González-Recio O, Bedhiaf-Romdhani S. Random regression models to estimate genetic parameters for milk yield, fat, and protein contents in Tunisian Holsteins. J Anim Breed Genet. 2023;140:440–61. 36965122 10.1111/jbg.12770

[51] Mulder HA, Rönnegård L, Fikse WF, Veerkamp RF, Strandberg E. Estimation of genetic variance for macro-and micro-environmental sensitivity using double hierarchical generalized linear models. Genet Sel Evol. 2013;45:23. 23827014 10.1186/1297-9686-45-23 PMC3734065

[52] Garcia-Baccino CA, Marie-Etancelin C, Tortereau F, Marcon D, Weisbecker J-L, Legarra A. Detection of unrecorded environmental challenges in high-frequency recorded traits, and genetic determinism of resilience to challenge, with an application on feed intake in lambs. Genet Sel Evol. 2021;53:4. 33407067 10.1186/s12711-020-00595-x PMC7788967

[53] Togashi K, Lin C. Genetic modification of the lactation curve by bending the eigenvectors of the additive genetic random regression coefficient matrix. J Dairy Sci. 2007;90:5753–8. 18024769 10.3168/jds.2007-0363

[54] Togashi K, Lin C. Selection for milk production and persistency using eigenvectors of the random regression coefficient matrix. J Dairy Sci. 2006;89:4866–73. 17106117 10.3168/jds.S0022-0302(06)72535-8

[55] Young J, Doyle PT, Booth P. Strip grazing to control wool growth rate of sheep grazing green annual pastures. Aust J Exp Agric. 1999;39:247–58.

[56] Collins J, Chaikin M. Structural and non-structural effects in the observed stress-strain curve for wet wool fibres. J Text Inst. 1968;59:379–400.

[57] Sanchez-Molano E, Kapsona VV, Ilska JJ, Desire S, Conington J, Mucha S, Banos G. Genetic analysis of novel phenotypes for farm animal resilience to weather variability. BMC Genet. 2019;20:84. 31718555 10.1186/s12863-019-0787-z PMC6849266

[58] Moncur VS, Hardie LC, Dechow CD. Genetic analysis of daily milk yield variability in Holstein dairy cattle in an experimental herd. Livest Sci. 2021;244: 104397.

[59] Cobuci JA, Euclydes RF, Costa CN, Torres RdA, Lopes PS, Pereira CS. Genetic evaluation for persistency of lactation in Holstein cows using a random regression model. Genet Mol Biol. 2007;30:349–55.

[60] Hynd P. Responses of sheep differing in fibre length to diameter ratio to nutritional change. Proc Aust Soc Anim Prod. 1992;19:152.

[61] Schlink A, Mata G, Lewis R. Consequences of differing wool growth rates on staple strength of merino wethers with divergent staple strengths. Wool Tech Sheep Breed. 1998;46:271–85.

