# Supplementary figures and images for: Analysis of the genetic variance of fibre diameter measured along the wool staple for use as a potential indicator of resilience in sheep
Source: Genet Sel Evol. 2024 Aug 6;56:57. doi: 10.1186/s12711-024-00924-4 (PMC11536905; doi:10.1186/s12711-024-00924-4)

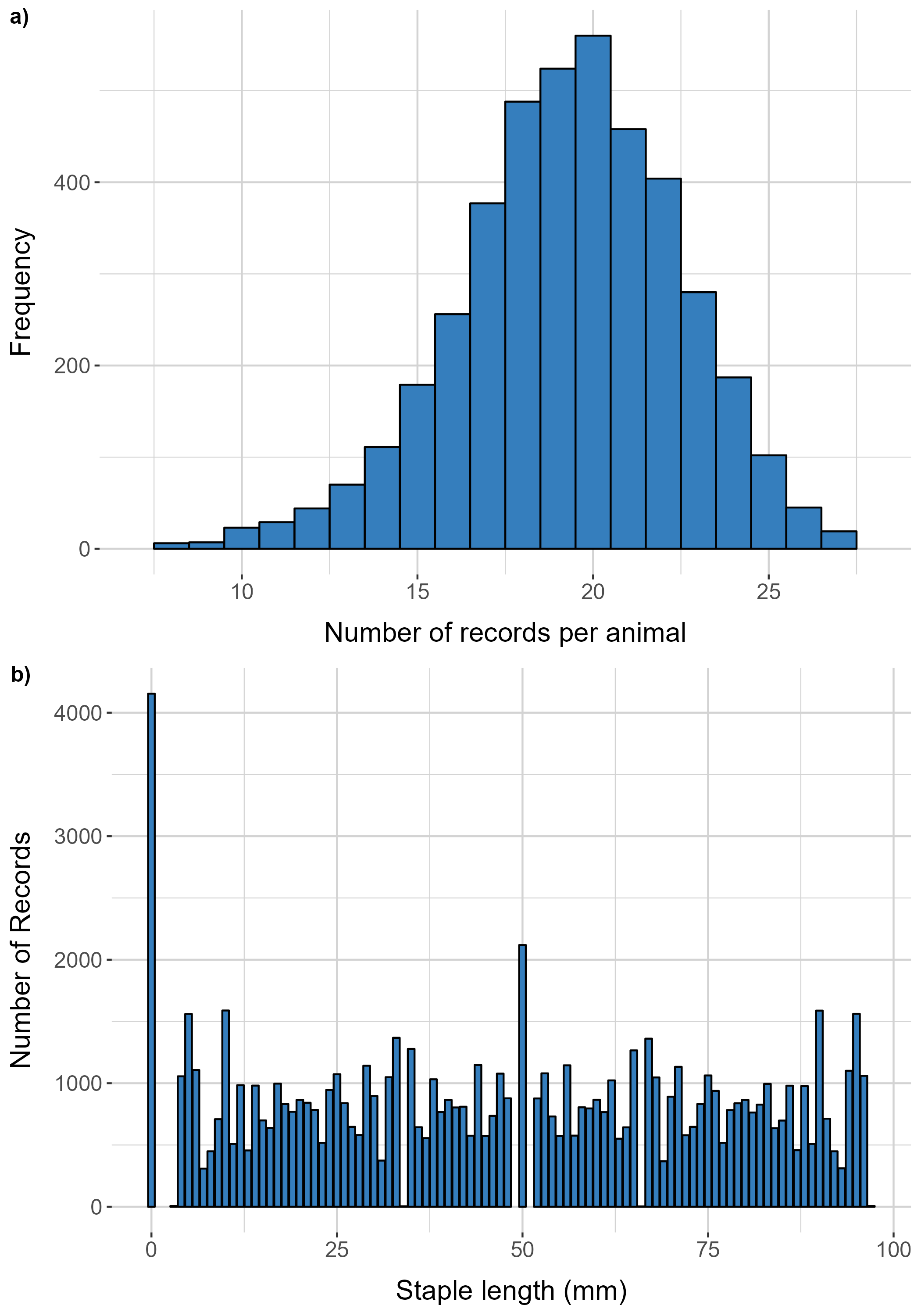

Supplement: Supplementary file 1 — Additional file 1: Figure S1. The distribution of the number of fibre diameter records per animaland the distribution of the standardised fibre diameter records at different staple lengths. [file 12711_2024_924_MOESM1_ESM.png]

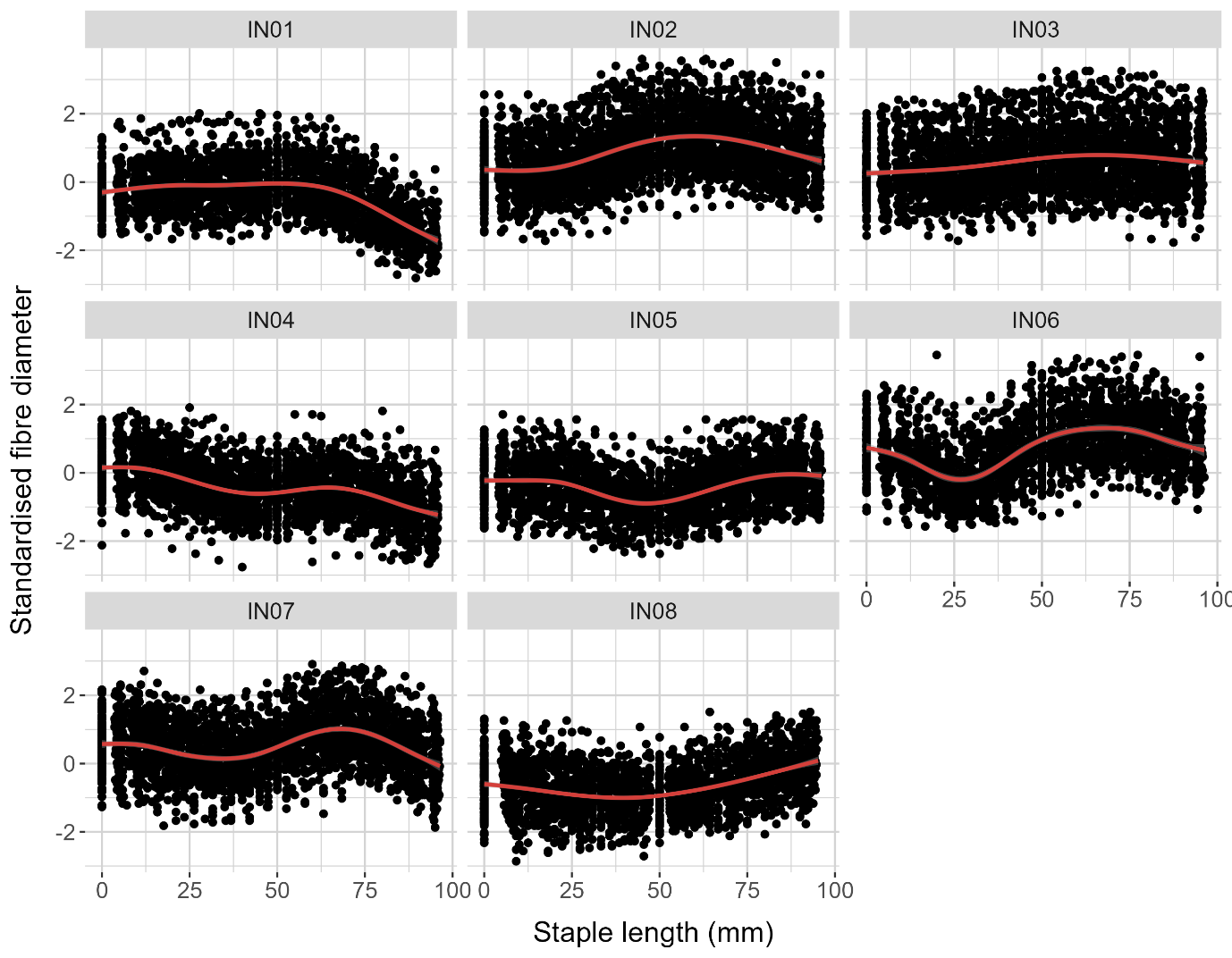

Supplement: Supplementary file 3 — Additional file 3: Figure S2. Example of the standardised fibre diameter values plotted along the wool staple for Information Nucleus Flocks 1 to 8 during the 2011 year of recording. The red line indicates the average curve estimates for each flock year combination. [file 12711_2024_924_MOESM3_ESM.png]

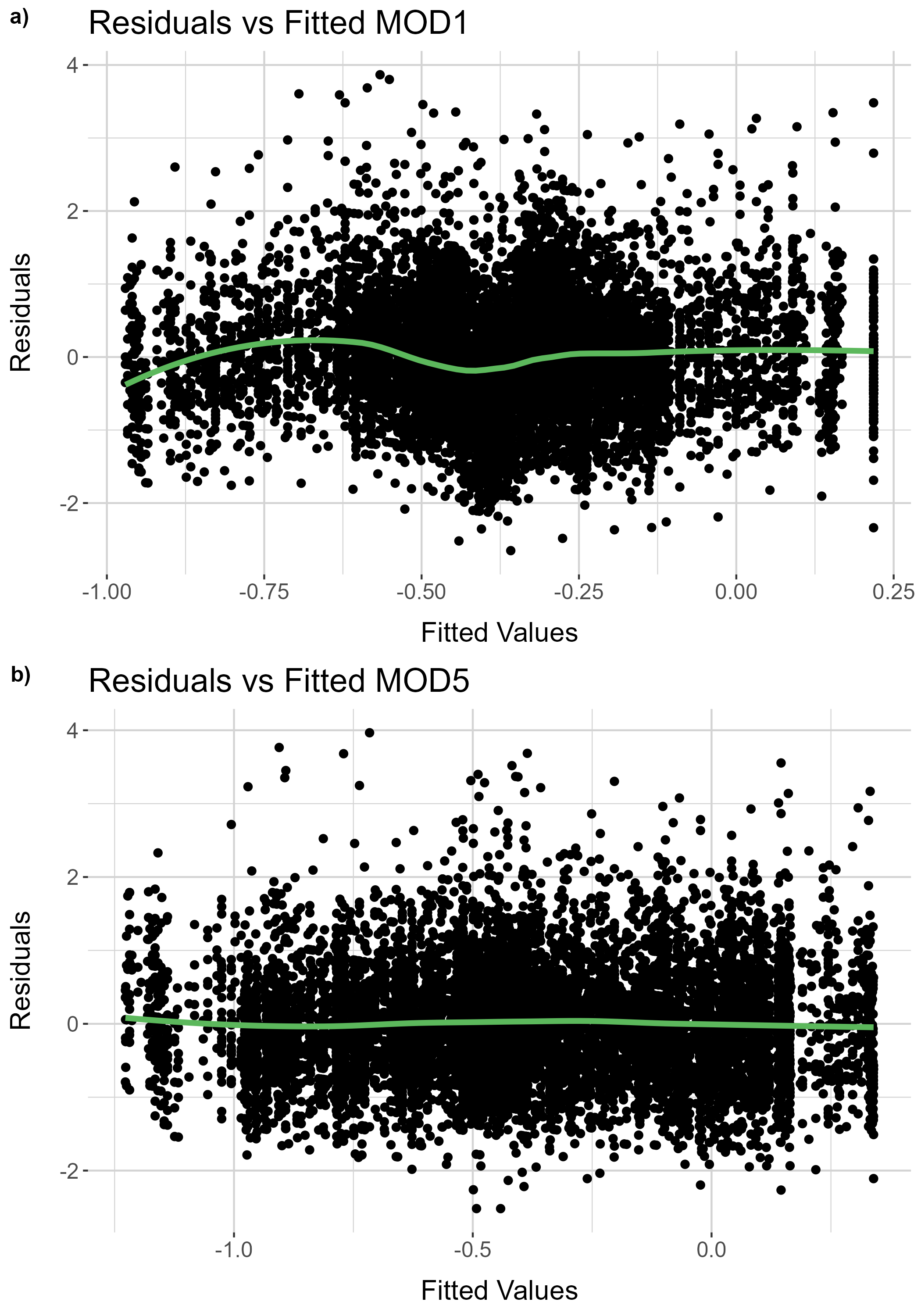

Supplement: Supplementary file 5 — Additional file 5: Figure S3. Residual versus fitted plots of standardised fibre diameter from A) MOD1 and B) MOD5 which test the order of Legendre polynomial applied to the fixed regression curve. In a well-behaved linear regression model, the residuals should exhibit constant varianceacross all levels of the fitted values. The green line represents the locally weighted scatterplot smoothing linewhich helps visualise whether the spread of the residuals remains roughly constant as the fitted values change. If the spread of the residuals widens or narrows systemically as the fitted values increase or decrease it suggests heteroscedasticity which violates the assumption of constant variance. [file 12711_2024_924_MOESM5_ESM.png]

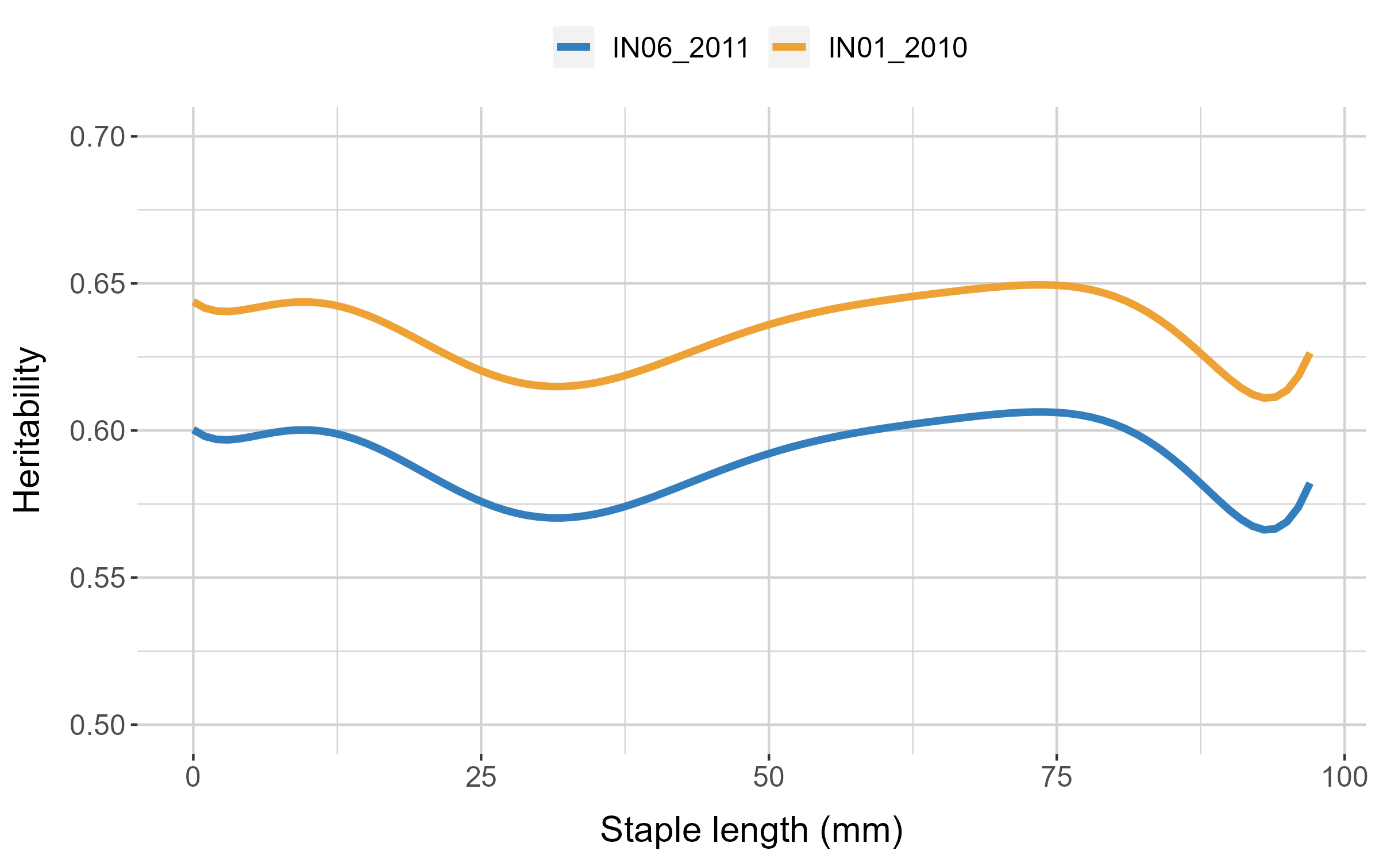

Supplement: Supplementary file 6 — Additional file 6: Figure S4. Heritability estimates of standardised fibre diameter measured along the wool staple for the flock years with the lowestand highestresidual variance estimates. The approximate standard error of the heritability estimates ranged between 0.089 and 0.095 for each flock-year. [file 12711_2024_924_MOESM6_ESM.png]
