# Supplementary material for: Analysis of the genetic variance of fibre diameter measured along the wool staple for use as a potential indicator of resilience in sheep
Source: Genet Sel Evol. 2024 Aug 6;56:57. doi: 10.1186/s12711-024-00924-4 (PMC11536905; doi:10.1186/s12711-024-00924-4)
Supplement: Supplementary file 2 — Additional file 2: Table S1. Structure of the dataset used to evaluate standardised fibre diameter along the wool staple at each of the eight Information Nucleus Flock sites. [file 12711_2024_924_MOESM2_ESM.docx]

**Additional file 2: Table S1.Structure of the dataset used to evaluate standardised fibre diameter along the wool staple at each of the eight Information Nucleus Flocks.**

| **Flock** | **Number of animals** | **Number of years** | **Average number of animals per flock years** | **Mean raw fibre diameter (µm)** | **Number of sires used** |
| --- | --- | --- | --- | --- | --- |
| 1 | 752 | 5 | 150.4 | 17.5 | 161 |
| 2 | 410 | 4 | 102.5 | 19.8 | 73 |
| 3 | 482 | 5 | 96.4 | 19.4 | 89 |
| 4 | 459 | 5 | 91.8 | 17.8 | 90 |
| 5 | 363 | 4 | 90.8 | 17.6 | 76 |
| 6 | 394 | 5 | 78.8 | 19.2 | 84 |
| 7 | 610 | 5 | 122.0 | 19.1 | 95 |
| 8 | 711 | 5 | 142.2 | 18.1 | 158 |
| **Total** | **4181** | **38** |  |  | **176^1^** |

^1^Unique sires
