# Supplementary material for: Analysis of the genetic variance of fibre diameter measured along the wool staple for use as a potential indicator of resilience in sheep
Source: Genet Sel Evol. 2024 Aug 6;56:57. doi: 10.1186/s12711-024-00924-4 (PMC11536905; doi:10.1186/s12711-024-00924-4)
Supplement: Supplementary file 4 — Additional file 4: Table S2. Bayesian Information Criteria, F statistics and partial F-test results from fixed regression of models containing Legendre polynomials of orders 1 to 6. [file 12711_2024_924_MOESM4_ESM.docx]

**Additional file 4: Table S2. Bayesian information criteria (BIC), F statistics and Partial F-test results from fixed regression of models containing Legendre polynomials of orders 1 to 8.**

| **Model** | **BIC** | **F statistic** | **Partial F-test^1^** |
| --- | --- | --- | --- |
| MOD1 | 227242.5 | 80.7 | - |
| MOD2 | 224658.7 | 71.1 | (1-2) *** |
| MOD3 | 222428.4 | 18.7 | (2-3) *** |
| MOD4 | 222150.0 | 15.0 | (3-4) ** |
| MOD5 | **222001.1** | **4.5** | (4-5) *** |
| MOD6 | 222267.1 | 11.3 | (5-6) NS (0.10) |

^1^ Where *** *P* = <0.001, ** 0.01, * 0.05, NS = non-significant with *P*-value in brackets.

^2^ Values in boldface type indicate the best model.
